# Complement Activation and STAT4 Expression Are Associated with Early Inflammation in Diabetic Wounds

**DOI:** 10.1371/journal.pone.0170500

**Published:** 2017-01-20

**Authors:** Kenji M. Cunnion, Neel K. Krishna, Haree K. Pallera, Angela Pineros-Fernandez, Magdielis Gregory Rivera, Pamela S. Hair, Brittany P. Lassiter, Ryan Huyck, Mary A. Clements, Antoinette F. Hood, George T. Rodeheaver, Patrick S. Cottler, Jerry L. Nadler, Anca D. Dobrian

**Affiliations:** 1 Department of Pediatrics, Eastern Virginia Medical School, Norfolk, VA, United States of America; 2 Department of Microbiology and Molecular Cell Biology, Eastern Virginia Medical School, Norfolk, VA, United States of America; 3 Children’s Specialty Group, Children’s Hospital of The King’s Daughters, Norfolk, VA, United States of America; 4 Department of Plastic Surgery, University of Virginia, Charlottesville, VA, United States of America; 5 Department of Physiological Sciences, Eastern Virginia Medical School, Norfolk, VA, United States of America; 6 Department of Dermatology, Eastern Virginia Medical School, Norfolk, VA, United States of America; 7 Department of Pathology, Eastern Virginia Medical School, Norfolk, VA, United States of America; 8 Department of Internal Medicine, Strelitz Diabetes Center, Eastern Virginia Medical School, Norfolk, VA, United States of America; Cedars-Sinai Medical Center, UNITED STATES

## Abstract

Diabetic non-healing wounds are a major clinical problem. The mechanisms leading to poor wound healing in diabetes are multifactorial but unresolved inflammation may be a major contributing factor. The complement system (CS) is the most potent inflammatory cascade in humans and contributes to poor wound healing in animal models. Signal transducer and activator of transcription 4 (STAT4) is a transcription factor expressed in immune and adipose cells and contributes to upregulation of some inflammatory chemokines and cytokines. Persistent CS and STAT4 expression in diabetic wounds may thus contribute to chronic inflammation and delayed healing. The purpose of this study was to characterize CS and STAT4 in early diabetic wounds using db/db mice as a diabetic skin wound model. The CS was found to be activated early in the diabetic wounds as demonstrated by increased anaphylatoxin C5a in wound fluid and C3-fragment deposition by immunostaining. These changes were associated with a 76% increase in nucleated cells in the wounds of db/db mice vs. controls. The novel classical CS inhibitor, Peptide Inhibitor of Complement C1 (PIC1) reduced inflammation when added directly or saturated in an acellular skin scaffold, as reflected by reduced CS components and leukocyte infiltration. A significant increase in expression of STAT4 and the downstream macrophage chemokine CCL2 and its receptor CCR2 were also found in the early wounds of db/db mice compared to non-diabetic controls. These studies provide evidence for two new promising targets to reduce unresolved inflammation and to improve healing of diabetic skin wounds.

## Introduction

Diabetes affects an estimated 415 million humans worldwide leading to a multitude of complications resulting in increased morbidity and mortality[1]. Approximately 15% of individuals who live with diabetes will develop foot ulcers due to non-healing cutaneous wounds, and 84% of this population will end up with lower-leg amputations[2]. Normal wound healing is a highly regulated dynamic series of events involving interfaces of blood cells, proteins, proteases, growth factors, cytokines, extracellular matrix components, and stromal cells. There are three overlapping phases that define classical wound healing; (1) inflammatory phase, (2) proliferative phase, and (3) remodeling and maturation phase[3]. The inflammatory phase is the first phase and is characterized by hemostasis and inflammation through the formation of a clot and release of several chemokines from platelets[3]. Dysregulated clot formation is one of the underlying mechanisms that contribute to abnormal wound healing in diabetic patients along with poor re-vascularization, repeated bacterial infections and unresolved inflammation[4]. This dysregulation is amplified due to hypoxia in diabetic patients caused by peripheral vascular disease[5].

Diabetic wounds are slow to heal and can therefore become chronic and can develop further complications such as infection potentially resulting in amputation. While an acute inflammatory response may be beneficial and participates in initiation of healing, unresolved inflammation prevents timely healing in diabetic wounds[4,6]. Part of the unresolved inflammation may be related to increased systemic inflammation associated with diabetes itself[7,8]. Aspects of inflammation known to be associated with abnormal wound healing in diabetes include prolonged presence of neutrophils and macrophages in the unhealed wound[9,10]. Interestingly, despite their abundance, neutrophils from diabetic patients have reduced migratory capacity and macrophages have a persistent pro-inflammatory phenotype (M1)[11,12]. Currently this is an evolving field as new aspects of the inflammatory response and diabetic wounds are explored. To date, the potential contributions of complement-mediated inflammation and STAT4-mediated inflammation remain largely unknown in the context of diabetic wounds.

The complement system is the most potent inflammatory cascade in humans[13,14]. Complement activation is notable for a rapid onset occurring in seconds and amplification of the signal at each step of the cascade[14]. Complement activation leads to the generation of many potent effectors including the anaphylatoxin C5a, which is important for leukocyte recruitment and activation, and C3-fragments C3b and iC3b that are major opsonins targeting leukocyte attack. Complement can be activated by the classical, lectin or alternative pathways, but most commonly activation is triggered by the classical or lectin pathways with the alternative pathway functioning as a positive-feedback loop[15]. Complement activation occurs in skin wounds and has been shown to contribute to altered wound healing in several models including burn wounds[16–19]. It is likely that complement plays multiple roles in a wound including innate immune protection against infection as well as potentially inhibiting healing if activation is dysregulated. Dysregulated complement activation clearly plays a prominent role in many inflammatory diseases where it contributes to host tissue damage[20]. New information also suggests that diabetes may contribute to dysregulation of the complement system[21,22]. We have previously shown that hyperglycemia alters complement mediated control of *S*. *aureus* infection[23,24]. Together, these emerging concepts and knowledge suggest that complement activation may be dysregulated in diabetic wounds. Peptide Inhibitor of Complement C1 (PIC1), is a peptide that inhibits enzymatic activation of the first component of the classical complement pathway C1[25,26], providing a proof-of-concept tool to test classical complement pathway activation.

Signal transducer and activator of transcription 4 (STAT4) has been associated with inflammation in both Type 1 and Type 2 diabetes and is primarily activated by IL-12 to promote cytotoxic responses and T helper 1 cell differentiation[27]. It is also a known mediator of IFNγ production by T cells and is associated with Th1 differentiation in mice and humans[28,29]. We have shown that STAT4 deficiency or inhibition protects from cytokine induced islet cell death and disruption of its expression prevents spontaneous development of Type 1 diabetes in mice[30–32]. We have also reported that STAT4 is a mediator of meta-inflammation and insulin resistance in adipose tissue in obesity[33] and has been shown to have increased expression in injured lesions in blood vessels of diabetic rats[34]. Mice deficient in STAT4 showed reduced CD8+ numbers in adipose tissue along with increased CD206+ M2 macrophage polarization and reduced IL12a in adipose tissue and islets due to decreases in M1macrophage numbers compared to STAT4 sufficient mice[33,35]. Diabetic wounds show persistent activation of macrophages and increased pro-inflammatory cytokines such as IFNγ, TNFα, IL1β, and IL-12 from macrophages biased towards the M1 inflammatory activation state. This persistent inflammatory state, complicates wound healing[36]. Therefore, persistent STAT4 expression and activation in diabetic wounds may contribute to chronic inflammation and delayed healing, and its relationship to complement is currently not well defined in the context of early and chronic stages of wound healing.

## Materials and Methods

### Ethics statement

Mouse experiments were performed under a protocol approved by the University of Virginia Institutional Animal Care and Use Committee in accordance with the National Institutes of Health’s Guide for the Care and Use of Laboratory Animals. Mice were housed in an AAALAC-accredited facility. All procedures were performed under anesthesia and all efforts were made to minimize pain or suffering.

### Mouse strains

Genetically diabetic C57BL/Ks db/db male mice, 10–12 weeks of age and heterozygous age-matched controls were used in all experiments. Diabetic mice exhibited glucose levels >300mg/dl at the time of wound generation.

### Mouse experiments

All animals were anesthetized using a mixture of isoflurane and oxygen. The hair on their back was shaved and depilated, and then the skin was cleaned with povidone iodine solution and alcohol. Under sterile conditions, two full-thickness excisional wounds of 8mm in diameter were generated bilaterally on the back of the mice, approximately 5mm from the spine, with a 6mm sterile biopsy punch.

### Early inflammation in wounds of diabetic mice

For the experiments comparing db/db with heterozygous mice without an immunomodulatory intervention, 18 mice were used in each group. A 6 mm filter paper disk (Whatman #52, GE Healthcare, PA) was placed into the wound followed by dressing with Tegaderm™ (3M, MN) and sealed with benzoin. Each animal was wrapped in a bolster dressing to keep the dressing in place. db/db and heterozygous mice in groups of three were anesthetized with isoflurane at the designated time points (0, 2, 4, 8, 24, and 48 hours) from the time of wounding. The filter paper disks were recovered and flash frozen. Blood (0.5 ml) was collected by cardiac puncture under sedation, into K_2_EDTA vials and centrifuged to recover the plasma. Euthanasia was then performed by anesthetic overdose (Pentobarbital and phenytoin). Wounds were excised “en bloc” and equally bisected in the sagittal plane, with half of each wound immediately fixed by submersion in 10% buffered formalin for histological evaluation and the other half of each wound placed into Trizol reagent (Thermo Fisher, MA), vortexed and flash frozen for subsequent PCR analysis.

### Diabetic mouse wounds with complement inhibitor PIC1 in gel

In another series of experiments, the wounds of db/db and heterozygous mice were treated with a gel vehicle or with HEC (hydroxyethyl cellulose) with or without the complement inhibitor PIC1 (n = 12 mice/experimental group). After anesthesia and wounding, animals received a topical application 0.1 ml of 2% HEC gel ± PIC1 (20 mg/ml, final concentration). Tegaderm™ and bolster dressings were placed as described above. At designated time points (4, 8, 24, and 48 hours) 3 mice per group were anesthetized, their dressings were removed, and a 6 mm filter paper disk (Whatman #52) was placed in the wound for 5 minutes, retrieved and frozen. Blood was obtained via cardiac puncture in heparinized tubes for plasma and the wounds were excised and processed for analysis as described above. Animals were then euthanized using anesthetic overdose (Pentobarbital and phenytoin).

### Diabetic mouse wounds with complement inhibitor PIC1 in a skin scaffold

The use of an acellular dermal matrix has been well documented in the treatment of hard to heal diabetic wounds[37]. db/db mice received a decellularized skin scaffold (DermACELL™, LifeNet Health, VA) that was saturated with the complement inhibitor PIC1 or the vehicle control and placed into the wounds (n = 6 mice/group). The purpose of this approach was to evaluate the effectiveness of the matrix as a carrier of the inhibitor, as well as a potential scaffold to promote healing. Therefore, only db/db mice were used to evaluate the effects of PIC1 and the scaffold in diabetic wound healing. Prior to placement in the wounds, the scaffolds were prepared by extrusion of the storage buffer under pressure followed by saturation with 0.3 ml of PIC1 (to an approximate final concentration of 20 mg/ml) or vehicle (saline). After anesthesia and wounding, an 8 mm disk of the scaffold (± PIC1) was placed into each bilateral skin wound and sutured into place. The scaffold was covered with Xeroform™ (DeRoyal, TN) and then Tegaderm™ and the bolster dressing. At 24, 48 and 72 hours after surgery, all mice were anesthetized again with isoflurane/oxygen mix, their bandages removed and the DermACELL™ was injected with 0.12 ml of PIC1 (40 mg/ml) or vehicle after which the mice were re-bandaged and allowed to recover. At day 14 after wounding, mice were anesthetized with isoflurane the wounds were photographed and blood was collected by cardiac puncture. Animals were then euthanized via anesthetic overdose (Pentobarbital and phenytoin) and wound tissues were recovered and processed as described above.

### Complement C5a ELISA

Frozen filter paper disks were thawed and placed in an individual well of a 96 well plate with 50 μl of 1% Triton X-100. The plate was gently shaken for 1 hour at room temperature and then fluid absorbed in the filter paper was expressed by compression into the well. The liquid samples were then measured using the mouse Complement C5a ELISA kit (R&D Systems, MN). Values for samples collected from the left and right wound of each mouse were averaged.

### Real-time quantitative PCR

Samples of wound tissue were homogenized in Tri-Reagent using a Tissumizer rotor-stator homogenizer (Teledyne Tekmar, OH). RNA was isolated by either chloroform phase separation with an Ambion Ribopure kit (Life Technologies, NY), or directly from Tri-Reagent with a Direct-Zol miniprep plus kit (Zymo Research, CA), and subsequently reverse transcribed into cDNA using iScript supermix (Bio-Rad, CA). Genes of interest were then assayed by qPCR using mouse TaqMan probes for STAT4 (Mm00448890_m1) Ccl2 (Mm00441242_m1) and Ccr2 (Mm00438270_m1) (Life Technologies, NY) with JumpStart Taq polymerase (Sigma-Aldrich, MO), 3 mM MgCl_2_, and 200 μ M dNTPs (Promega, WI) using a Bio-Rad CFX96 C1000 thermocycler. A standard TaqMan cycling protocol was performed, consisting of a 10 min 95°C hold, followed by 40 rounds of 15 sec at 95°C and 1 min at 60°C. 18s expression was used as a housekeeping control for data normalization.

### Preparation of tissue histology

Skin tissues were embedded and sectioned to make slides for histological evaluation. Histological sections were subjected to routine de-paraffination and hydration before antigen retrieval using sodium citrate (10 mM sodium citrate, 0.05% Tween 20, pH 6.0). Hematoxylin and eosin staining was performed per routine.

C3-fragment staining was performed after blocking with 10% normal goat serum in 1× PBS (NGS-PBS) for 1 hour. The sections were then probed for C3-fragments, including C3a and opsonic forms like C3b/iC3b, with a chicken anti-C3/C3a antibody (Abcam, MA) that recognizes rat and mouse C3/C3a. Secondary staining was performed with a goat anti-chicken IgG (H+L) labeled with Alexa Fluor 488 (AF 488) (Life Technologies, NY); the slides were mounted with a Vectashield® anti-fade mounting medium containing DAPI (Vector Laboratories, CA) for visualization of nuclei. STAT4 was detected by immunofluorescence using a polyclonal anti-STAT4 antibody from Santa Cruz Biotechnology (sc-486) and a secondary fluorescent antibody DyLight 549 (Vector Laboratories, CA). Nuclei were stained using DAPI. STAT4 expression was evaluated in a blind fashion by 4 independent observers. The dermal and adipose tissue areas were analyzed separately. Sections were graded on a scale of 0–3 according to the overall signal on each of the sections as following: 0 = 0–5%; 1 = 5–25%; 2 = 25–50%; 3>50%. The sampling protocol included 5–8 sections from 3 mice/group. Results from the four observers were averaged and expressed as average +/- SD.

### Light microscopy and leukocyte infiltration analysis

Leukocyte infiltration was evaluated for the skin wounds by H&E stained slides or by DAPI stained slides. H&E stained slides were evaluated for inflammatory infiltrate by Dr. Hood, a Board certified Dermatopathologist. Dr. Hood analyzed the H&E slides in a blinded manner evaluating leukocyte infiltration at both the edges and base of the skin wounds. Leukocyte inflammatory infiltrate was graded on a clinical scale of 0–4 and averaged for the edge and base of each wound.

### Fluorescence microscopy

Images of AF 488 stained C3-fragments and DAPI stained nucleated cells were taken with an Olympus DP70 digital camera mounted on an Olympus BX50 microscope. The images were cropped using Adobe Photoshop CS5 to isolate the subcutaneous area of the tissue. C3-fragment expression and nucleated cell counts were measured using ImageJ. C3-fragment levels were quantified by integrated intensity using the color threshold option to reduce the background signals. Nucleated cell counts were quantified by converting the image into an 8-bit binary image and measuring the particle count.

### Classical pathway inhibition with peptide inhibitor of complement C1 (PIC1)

PIC1 (i.e., PA-dPEG24)[25], identified and characterized by our group [26], is an inhibitor of antibody-initiated classical complement pathway activation that blocks the enzymatic activity of the first component of the classical pathway C1. PIC1 was synthesized as a lyophilized powder (>90% purity) by New England Peptide (MA).

### PIC1 in HEC gel

4% hydroxyethyl cellulose (HEC) gel was first prepared by adding HEC powder (TCI America, OR) into sterile water, boiled for 5 minutes with stirring, and then cooled with stirring until the gel became semi-solid at room temperature. An equal amount of filtered sterile buffer (0.9% NaCl, 10mM Na_2_PO_4_, pH 7.4), containing PIC1 at a concentration of 40mg/mL, was added to the cooling HEC gel to achieve a final concentration of 2% HEC and 20 mg/ml PIC1. Control groups contained equal parts HEC gel and buffer alone. 0.1mL of gel was added to each wound using a sterile syringe.

### Acellular skin scaffold

Acellular skin scaffold (DermACELL), derived from cadaveric human skin, was provided by LifeNet Health (VA). *In vitro* experiments were performed with PIC1 demonstrating that it was absorbed by the scaffold and released from the scaffold with functional inhibition of classical pathway complement activation. PIC1 was absorbed into the skin scaffold to an approximate 20 mg/ml final concentration.

### Statistical analysis

TaqMan qPCR data was analyzed using two factor ANOVA with post-hoc contrasts to analyze longitudinal results by genotype using the Real Statistics Resource Pack software (Release 4.6) add-on for Excel. Comparisons between ±PIC1 and immunohistochemistry data were analyzed by unpaired Student’s *t*-test.

## Results

### Complement effectors and inflammation in db/db mice

**Fig 1:**
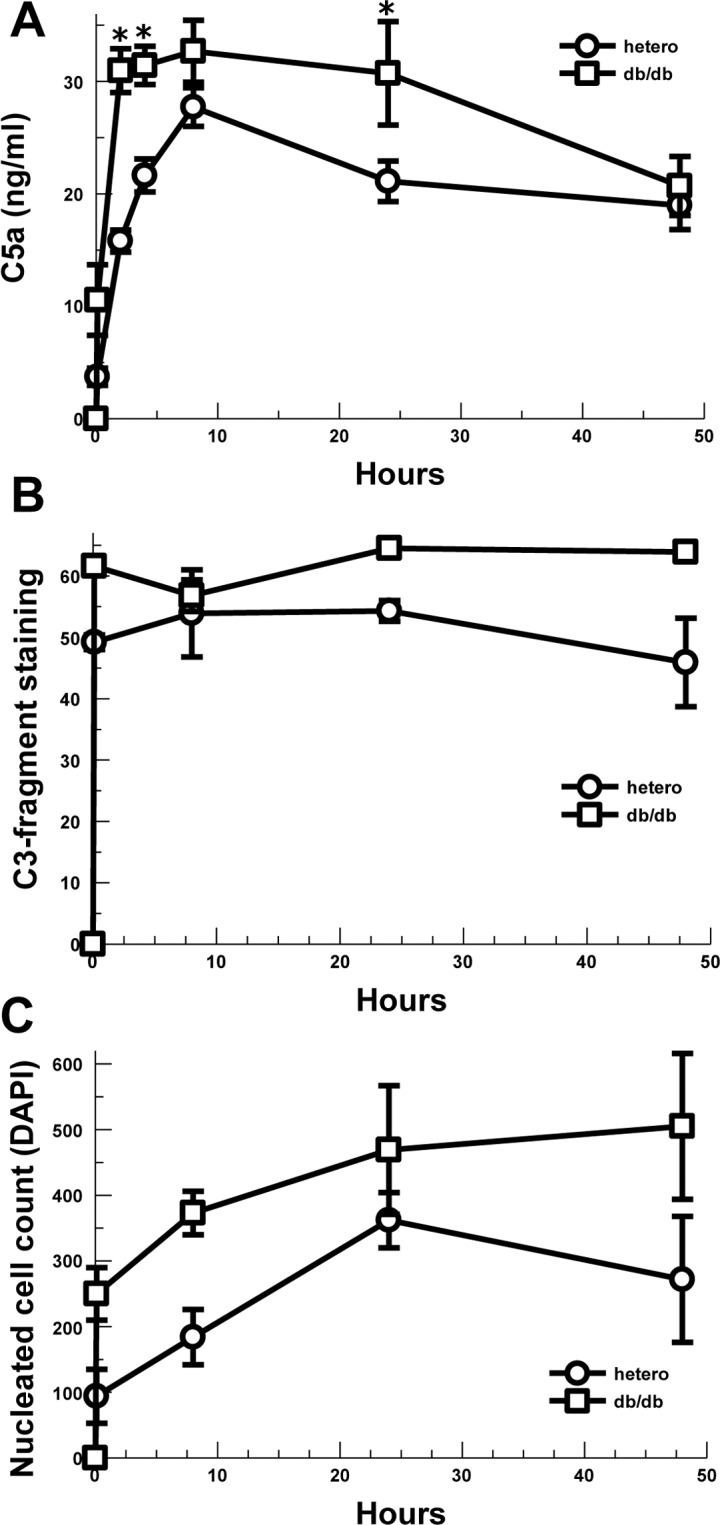
Complement activation and cellular inflammation for acute skin wounds in diabetic mice. (A) C5a concentration in wound beds of diabetic and control mice absorbed by filter paper and assayed by ELISA. Left and right wounds averaged for n = 3 mice in each group and time point: 10 min (P = 0.05) 2h (P = 0.002), 4h (P = 0.001), 24h (P = 0.05). * P ≤0.05 vs. hetero (control). (B) C3-fragment deposition (C3 opsonization) in the subcutaneous tissue at the edges of the wound beds of diabetic and control mice assayed by immunofluorescence (n = 2). (C) Nucleated cell infiltration into the subcutaneous tissue at the edges of the wound beds of diabetic and control mice assayed by DAPI fluorescence (n = 2). Data are means ± SEM.

In order to evaluate the generation of complement effectors in an animal model of diabetic skin wounds, we utilized db/db and heterozygous control mice. The anaphylatoxin C5a was assayed from wound fluid absorbed to filter paper from 10 minutes to 48 hours after wounding (Fig 1A). C5a concentration in the wound fluid of db/db mice was increased by 3-fold at 10 minutes (P = 0.05) and 2-fold at 2 hours (P = 0.002) compared with heterozygous mice. db/db mice also showed elevated C5a in wound fluid at 4 hours (P = 0.001) and 24 hours (P = 0.05) after wounding, compared to the heterozygous control animals.

C3-fragment (e.g. C3a, C3b, iC3b) staining of the wound tissues was noted to predominantly occur in the subcutaneous tissues at the edges of the wounds (S1 Fig). Two wounds from each mouse at each time point were available for quantitative analysis by Image J. C3-fragment deposition was noted at 10 minutes out to 48 hours consistent with rapid complement activation (Fig 1B). Because neither group showed a consistent increase or decrease over time, the data from all time points for each animal was combined for analysis. Overall the db/db mice showed a 22% increase in C3-fragment deposition in the subcutaneous tissues at the edge of the skin wounds compared with heterozygous controls (P = 0.003). Together these data suggest complement activation and effector generation occurs quickly after the skin wound is made, as expected, and the db/db mice show increased complement effectors in the subcutaneous tissue at the wound edge compared with controls.

In order to evaluate whether the generation of the anaphylatoxin and chemoattractant C5a was associated with leukocyte infiltration, we assessed the subcutaneous tissue at the wound edge for the numbers of nucleated cells using DAPI. The numbers of nucleated cells at the edge of the wound is shown in Fig 1C. Due to the small numbers of evaluable wounds in each group, the 10 minute through 48 hour data was combined for analysis revealing a 76% increase in nucleated cells for db/db mice compared with heterozygous controls (P = 0.039). Histological evaluation of H&E stained slides revealed that the vast majority of the leukocytes were neutrophils, consistent with acute inflammation (data not shown).

### Increased STAT4, CCL2 and CCR2 expression in diabetic wounds

**Fig 2:**
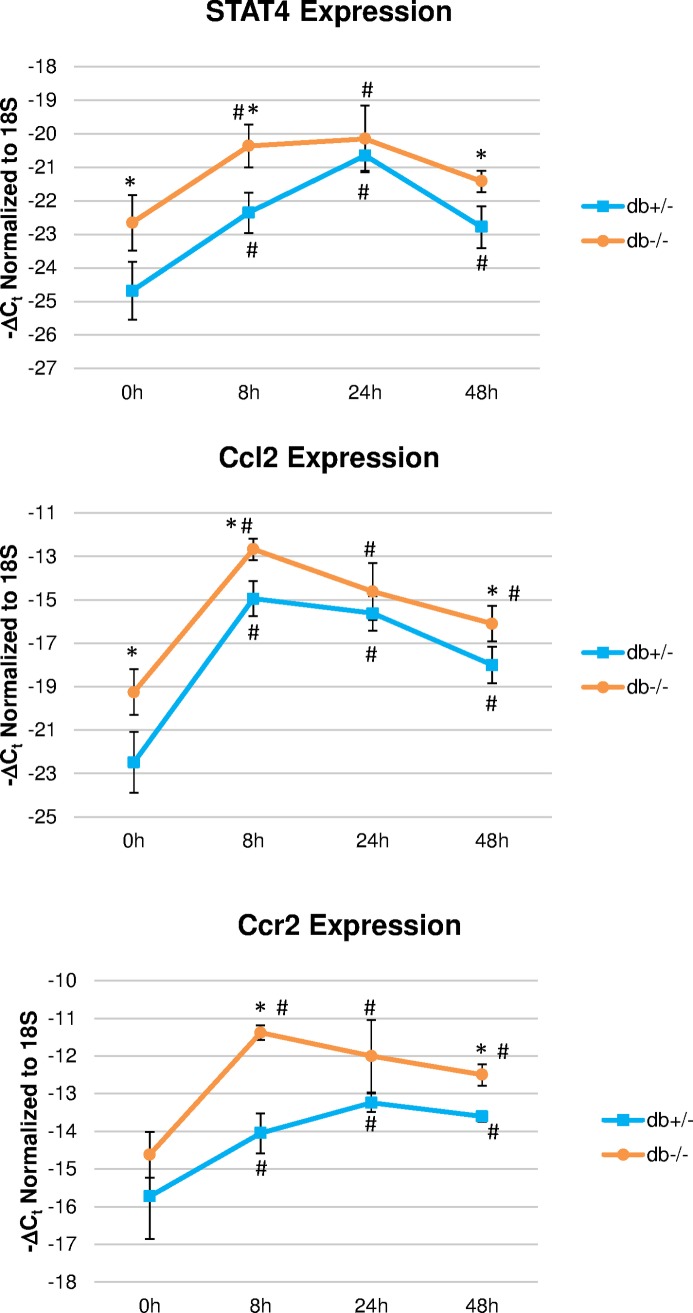
Longitudinal changes in gene expression of STAT4, Ccl2 and Ccr2 in diabetic db/db mice and controls. Wounds were excised at indicated time points and gene expression was measured by real-time PCR using Taqman probes. Results are expressed as 1/ΔCt and represent the average of two wounds/mouse from 3 mice/group +/-SEM. * P ≤0.05 vs. dbhet (control); # P ≤0.05 vs. t = 0 (approximately 10 minutes after wounding).

STAT4 is a key mediator of inflammation that induces Th1 polarization and IFNγ production. Previous studies showed that IFNγ is an important mediator of M1 polarization in wounds and prevents M2 macrophage polarization that is required for repair and healing[38]. CCL2 is a potent macrophage chemokine that is known to attract excessive number of macrophages and therefore may impair the resolution of the inflammatory phase. CCL2 exerts its chemoattractant functions primarily, although not exclusively, via binding to the CCR2 receptor. Expression of STAT4, CCL2 and CCR2 have not previously been described in the early inflammatory stage in db/db diabetic mice with skin wounds. Interestingly, we found that both Stat4 and Ccl2 gene expression were significantly higher at the time of wounding in db/db mice compared to heterozygous controls (Fig 2). In addition, Ccr2 expression was significantly increased at all time points compared to the time at wounding. Although the longitudinal pattern of expression was similar in both diabetic mice and in the heterozygous controls, expression of all three genes remained significantly higher in db/db mice compared to heterozygous controls at 8 and 48 hours after wounding (Fig 2).

**Fig 3:**
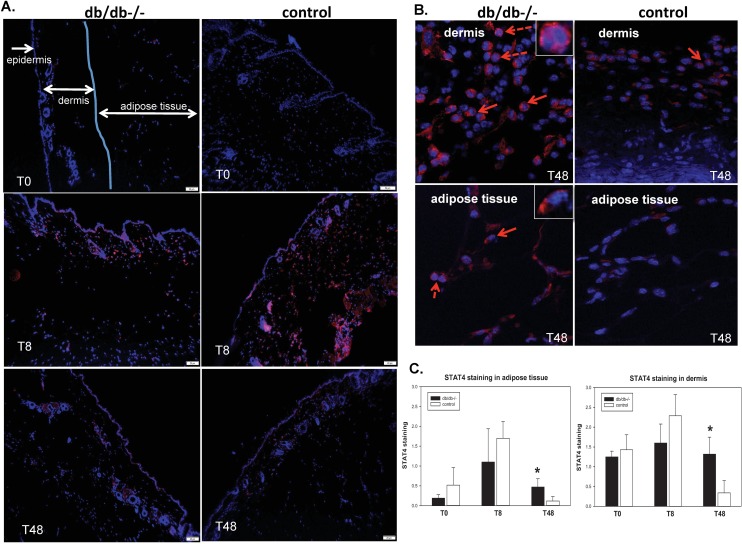
Immunohistochemistry showing longitudinal changes in expression of STAT4 in diabetic db/db and heterozygous control mice. **(A)** Representative micrographs showing STAT4 immunostaining (red) and nuclear staining using DAPI (blue) of formalin fixed paraffin embedded whole wounds of db/db-/- and control mice at the time of wounding (T0) and after 8 and 48 hours, respectively. Magnification: 100x; (B) High power (400x) images of the dermis (top) and adipose tissue (bottom) layers of the dbdb-/- and control mice at 48 hours post-wounding. Solid arrows indicate peri-nuclear localization of STAT4 and dashed arrows indicate nuclear localization. Insets represent higher magnification of the nuclear (top) or peri-nuclear (bottom) localization of the signal. (C) Grading of STAT4 staining in the dermis and dermal adipose tissue of diabetic db/db and control mice. A scale of “0” to”3” was used to quantify the abundance of staining. A number of 5–8 micrographs/section from n = 3 mice/group were graded by 4 independent observers in a blinded manner. * P ≤0.05.

Immunohistochemistry showed robust expression of STAT4 protein in the wounds of both diabetic mice and heterozygous controls that peaked at 8 hours after wounding (Fig 3A). STAT4 was not detected in the epidermal layer around the wound but expressed in both the dermal layer and the adjacent adipose tissue (Fig 3A). In both areas, expression of STAT4 was predominantly associated with the dermal layer and peaked at 8 hours after wounding in both db/db mice and heterozygous controls (Fig 3A and 3C). After 48 hours from the time of wounding, STAT4 expression decreased in the heterozygous control mice but was significantly higher in the db/db mice both in the dermis and in the adipose tissue, suggesting sustained elevated inflammation in the diabetic mice compared to heterozygous controls (Fig 3B and 3C). Functional STAT4 requires activation via phosphorylation and dimerization followed by nuclear transport. Although we were not successful in using a phosphoSTAT4 antibody, we did find STAT4 expression associated with the nuclei and, more frequently, peri-nuclear or cytoplastic localization (Fig 3B, arrows and high magnification insets). This indirectly indicates that a fraction of the STAT4 protein is indeed functionally active in the wounds.

### Classical complement pathway is increased in diabetic skin wounds

**Fig 4:**
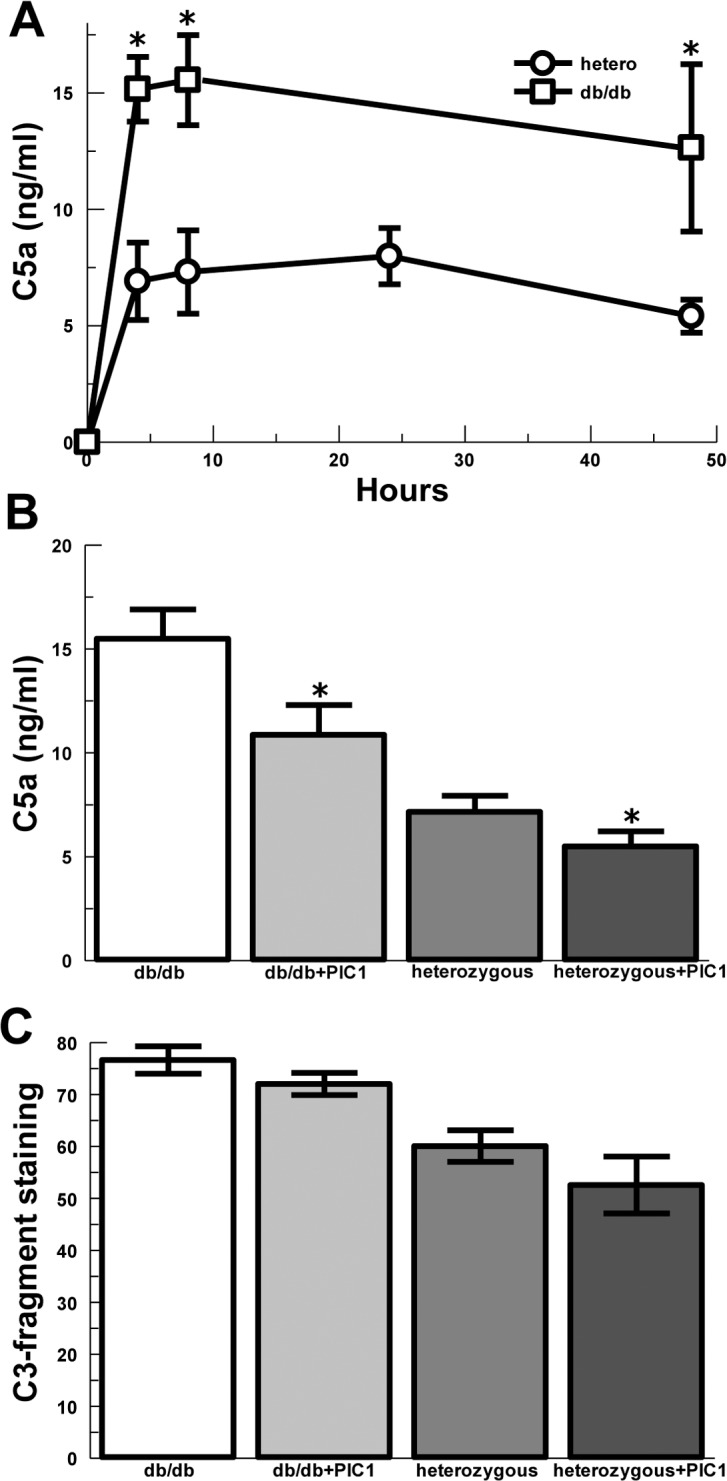
Complement effectors in acute skin wounds of diabetic and control mice covered with a gel ± complement inhibitor, PIC1. (A) C5a concentration in the wound beds of diabetic and heterozygous control mice absorbed by filter paper and assayed by ELISA. Left and right wounds averaged for n = 3 mice in each group and time point: 4H (P = 0.002), 8H (P = 0.007) and 48H (P = 0.05). (B) C5a concentration in the wound beds of diabetic and heterozygous control mice treated with vehicle control gel or PIC1 gel (combined time points). db/db ± PIC1 (P = 0.05). Heterozygous ± PIC1 (P = 0.01); data are means ± SEM. (C) C3-fragment deposition (C3 opsonization) in the subcutaneous tissue at the edges of the wound beds of diabetic and control mice treated with vehicle control gel or PIC1 gel (combined time points). db/db ± PIC1 (P = 0.09). Heterozygous ± PIC1 (P = 0.12), Data are means ± SEM. * P ≤0.05 vs. saline control.

In order to evaluate the contribution of classical complement pathway activation in the diabetic wound model, we utilized the classical complement pathway inhibitor, PIC1. A neutral gel, hydroxyethyl cellulose (HEC), was used as the vehicle for PIC1 delivery. The gel was placed into the wound and covered by a dressing. Filter paper disks were placed in the wounds for 5 minutes to capture wound fluid for C5a analysis. The trends for C5a concentrations in the wounds were again noted to be elevated for db/db mice compared with heterozygous controls demonstrating a 2-fold increase at 4 hours (P = 0.002), 8 hours (P = 0.007), and 48 hours (P = 0.05) (Fig 4A). Because complement activation occurs in seconds to minutes and little change occurred over 4–48 hours, the time points were combined to increase the robustness of statistical analysis. Overall, db/db mice treated with PIC1 in HEC gel showed a 24% decrease in C5a compared with HEC controls (P = 0.05) (Fig 4B) and heterozygous animals treated with PIC1 showed a 30% decrease in C5a compared with HEC only controls (P = 0.01). Deposition of C3-fragments (i.e., C3b/iC3b opsonization) in the subcutaneous tissues around the wounds (Fig 4C) showed non-significant trends towards decrease in the presence of PIC1 in HEC gel for both db/db (P = 0.09) and heterozygous mice (P = 0.12). These data suggest that classical pathway complement activation is occurring in or around the acute wound contributing to C5a generation and that inhibition of classical complement activation can decrease complement effector generation. PIC1 did not inhibit Ccl2 or Stat4 gene expression in db/db mice (S2 Fig), suggesting that a combination of PIC1 and an IL12/STAT4 pathway inhibitor could potentially yield superior results for reduction of sustained inflammation without compromising the very early beneficial inflammatory response.

**Fig 5:**
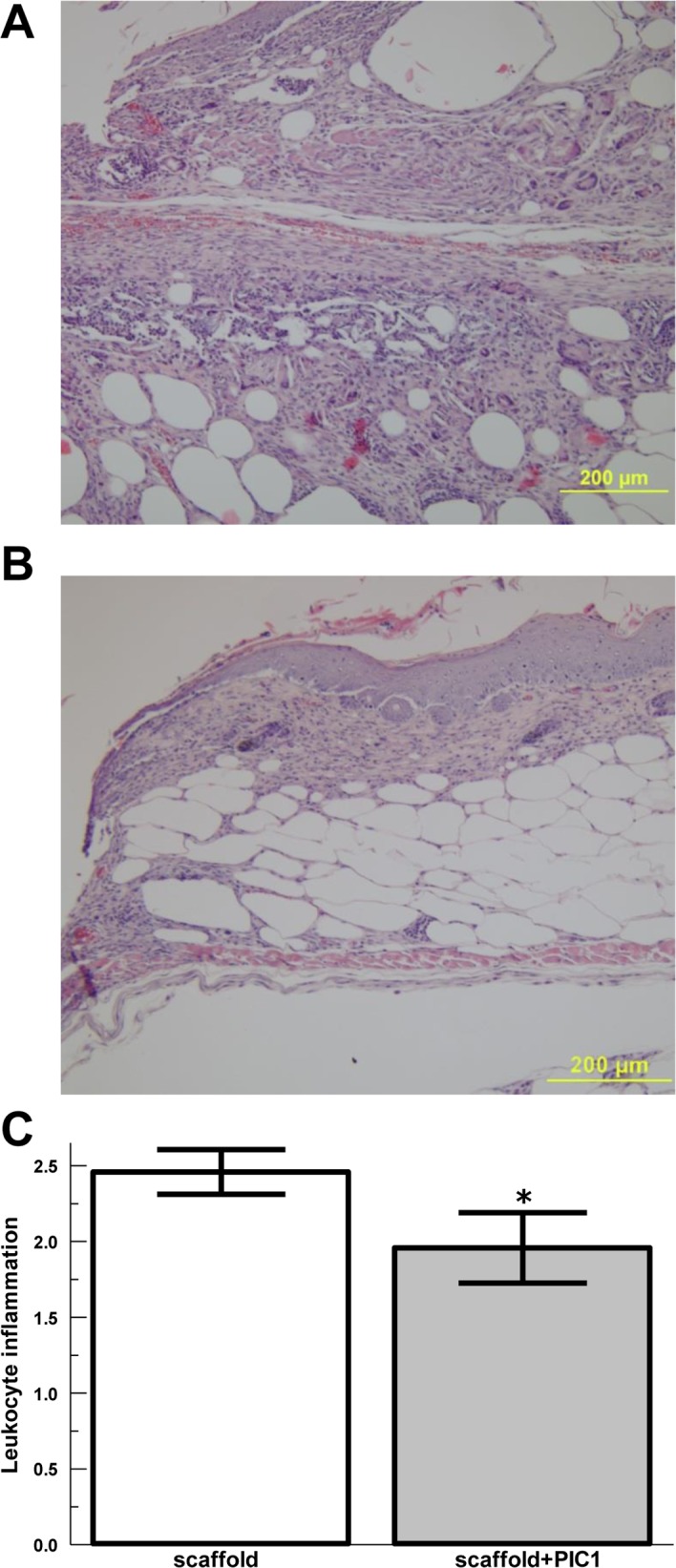
Analysis of leukocyte inflammation of wounds for diabetic mice treated with PIC1 at 14 days. Representative wound histology (H&E) for (A) control scaffold only mice, and (B) PIC1 impregnated skin scaffold. (C) Averaged inflammatory index of leukocytes (predominantly neutrophils) for db/db mice at day 14 post wounding for wounds treated with PIC1 complement inhibitor in a skin scaffold or control skin scaffold, n = 6 in each group. Data are means ± SEM. * P ≤0.05 vs. saline control.

In order to simulate a non-healing diabetic wound and evaluate later time points, we utilized an acellular skin scaffold to prevent closure of the wound by contraction and as a substrate for PIC1. Wounding of db/db mice was performed as above, but the acellular skin scaffold was placed into the wound and covered with a dressing. The skin scaffold was saturated with PIC1 or saline vehicle prior to placement and then PIC1 or saline were added to the scaffold at 24, 48 and 72 hours under the overlying dressing. Leukocyte infiltration into the tissues at the edges and base of the wounds at 14 days after wounding was assessed by a Dermatopathologist using a semi-quantitative clinical scale of 0–4 (Fig 5A and 5B). PIC1 treatment in the skin scaffold decreased leukocyte numbers, predominantly neutrophils, (Fig 5C) compared with saline control (P = 0.01). Wound closure was not different between the groups as expected due to stenting by the skin scaffold (data not shown).

## Discussion

Identifying new targets for improving poorly healing diabetic wounds represents an important unmet medical need. This study identifies two pathways of potential importance explaining excess inflammation in diabetic skin wounds. The hypothesis is that chronic, persistent inflammation is an important contributor to the pathogenesis of non-healing wounds in diabetic patients.

Previous studies have documented the delayed healing properties of diabetic wounds in a murine model. It has been shown that the time to complete closure of a 5mm diameter, splinted full thickness wound was doubled in db/db mice compared to heterozygous littermates (22.6 days vs. 12.7 days). Additionally, the healing kinetics of the two groups were statistically similar until day 8[39]. The complement cascade is rapidly activated in response to wounding[40], and it has been shown that mice deficient in C3 or C5 displayed accelerated wound healing in the early stages (days1-3) of normal wound healing[41]. Therefore, the critical aspect in determining the efficacy of complement as a therapeutic target was to evaluate the kinetics of C5 and C3a in the initial 48 hours post-wounding in both normal and diabetic wounds, and if inhibition of C1 could reestablish levels seen in normal wound healing.

These proof of concept data demonstrate increased concentrations of the anaphylatoxin C5a in the wound fluid of db/db mice compared with heterozygous controls. C5a is a strong chemotatic stimulus for leukocytes, especially neutrophils[42]. Increased C5a correlated with increased leukocyte infiltration, predominantly neutrophils, at the edges and base of the wounds of db/db mice compared with controls. Increased complement activation for the wounds of db/db mice was supported by demonstration of increased C3-fragments in the subcutaneous tissues compared with controls. These results suggest that diabetic wounds may produce excessive complement activation and C5a generation leading to increased neutrophil recruitment to the wound. Addition of the complement inhibitor PIC1 to the wound via a gel or skin scaffold was associated with decreased neutrophil migration to db/db wounds supporting the connection between complement activation in diabetic wounds and neutrophil inflammation.

The wound healing studies utilizing DermACELL as a carrier for the inhibitor into wounds demonstrated the effectiveness of the material as a carrier of the inhibitor, as well as a potential scaffold to promote healing. Therefore, only diabetic wounds were used to evaluate any effect in healing due to the application of the PIC1 complement inhibitor by quantifying the inflammatory cells in the wound bed. In this model wound closure was not used as a primary outcome due to the fact that the DermACELL remained in the wound bed, making re-epithelialization difficult to assess and wound closure through contracture could not be achieved. Results of our study did show that the PIC1 loaded into the DermACELL did reduce the number of inflammatory cells in the wound bed, demonstrating a potential effect for longer term healing.

In addition to complement activation leading to enhanced neutrophil recruitment to the wound, increased C3-fragments presence in the subcutaneous tissues likely illustrates increased deposition of C3b/iC3b opsonins on host tissues. C3-fragment opsonization targets cells for neutrophil attack [43] which is further enhanced by C5a serving as a stimulus for neutrophil degranulation. These effectors working in concert contribute to the release of toxic granule enzymes like neutrophil elastase and myeloperoxidase contributing to host tissue damage. In the setting of diabetic wounds, it is reasonable to argue that these mechanisms may contribute to abnormal wound healing.

We noted increased subcutaneous adipose tissue in the db/db mice, consistent with findings by prior investigators. The evolving understanding of diabetes attributes a major contribution to the disease pathogenesis by adipose tissue and adipocyte-mediated inflammation. Among other mechanisms of adipose-mediated inflammation in diabetes growing evidence supports the contribution of dysregulated complement activation [44]. Our findings appear to support the concept of diabetes causing dysregulated complement activation by demonstrating increased inflammatory complement effectors in the adipose tissue of diabetic mice. We speculate that that dysregulated complement activation in the diabetic adipose tissue bed may contribute to the abnormal wound healing seen in diabetic patients.

These results provide the foundation to determine more comprehensive effects of complement inhibition on diabetic wound healing. Future studies need to include dosing and delivery times as well as time to complete wound closure, to maximize the effect of PIC1 on diabetic healing.

STAT4 is primarily activated by IL-12, and has been implicated in both Type 1 and Type 2 diabetes. Disruption of STAT4 activation prevents spontaneous development of Type 1 diabetes in non-obese diabetic mice[31,32]. STAT4 has emerged as an important transcription factor regulating T-cell activation, macrophage inflammatory phenotype, insulin resistance and atherosclerosis[33,35]. STAT4 also regulates the expression of major cytokines and chemokines that regulate inflammatory cell migration into tissues such as the vasculature and adipose tissue[45,46]. STAT4 activation is downstream of Jak/Tyk kinases and STAT4 activation is seen in several cell types that could play a role in chronic wound inflammation. These include NK cells, macrophages, adipocytes, endothelial cells and dendritic cells[28,33,47]. We have recently shown that mice with STAT4 germline deletion demonstrate a bias towards CD206+ M2 “anti-inflammatory” macrophages, without a change in the total cell number[35]. Interleukin 12 (IL-12) is one of the major inducers of STAT4 activation, is produced by M1 “inflammatory” macrophages and has been implicated in impaired diabetic healing[36]. Also, other factors such as IFNγ have been shown to activate STAT4 in endothelial cells and elevated levels have been shown in lesions of injured blood vessels in diabetic animals[34]. Therefore, STAT4 is an important mediator of inflammation in immune cells and adipocytes in diabetes and obesity and sustained activation of STAT4 in skin wounds may contribute to perpetuation of inflammatory responses and impaired healing.

There has been very limited evaluation of STAT4 expression and its role in the skin. One very recent study showed immunostaining of STAT4 in the skin of patients with leprosy[48]. However, STAT4 was associated with pro-fibrotic processes in liver and kidney[49,50].

In the present study, we provide the first evidence for the presence and role of STAT4 in skin wounds of a typical type 2 diabetic mouse model. STAT4 expression was induced early in the wound and levels remained elevated up to 48 hours in the diabetic db/db mice compared to wounds in non-diabetic mice. Additional studies will be needed to evaluate further the particular cell type(s) expressing STAT4 as well as the full chronicity of expression. However, it was interesting that both STAT4 and the chemokine ligand/receptor pair CCL2/CCR2, that are linked to macrophage migration, were upregulated at time zero indicating a pre-activated inflammatory state in the dermis of the db/db mice. Importantly, STAT4 and the CCL2/CCR2 remained highly expressed in the db/db-/- mice 48 hours post-wounding suggesting early hyper-activation of an inflammatory pathway that can lead to delayed healing. Though the acute STAT4 expression profile was similar to C5a in both the normal and diabetic wounds (Figs 1 and 3), inhibition of complement through the application of PIC1 did not decrease STAT4 (S2 Fig). The role of STAT4 in participation to the chronic inflammatory state and poor resolution of healing of diabetic wounds will require further study. However, given the role of STAT4 in chronic inflammation in adipose tissue and other diabetic tissues, targeting STAT4 activation alone or in combination with complement blockage could provide a novel therapeutic approach to improve healing of diabetic wounds.

## Supporting Information

S1 Fig Fluorescent staining of C3-fragments. Representative micrographs showing complement C3-fragments deposited in the subcutaneous tissue at the skin wound edge for db/db mice and heterozygous controls.
(TIF)

S2 Fig Effect of PIC1 on Stat4 and Ccl2 gene expression in db/db diabetic mice. Wounds were excised at indicated time points and gene expression was measured by real-time PCR using Taqman probes. Results represent the average from 3 mice/group ±SEM. Differences between PIC1 treated and untreated wounds for each time point were analyzed using the unpaired t-test. The null hypothesis was rejected for a p-value <0.05.
(TIF)
