# Supplementary figures and images for: Complement Activation and STAT4 Expression Are Associated with Early Inflammation in Diabetic Wounds
Source: PLoS One. 2017 Jan 20;12(1):e0170500. doi: 10.1371/journal.pone.0170500 (PMC5249255; doi:10.1371/journal.pone.0170500)

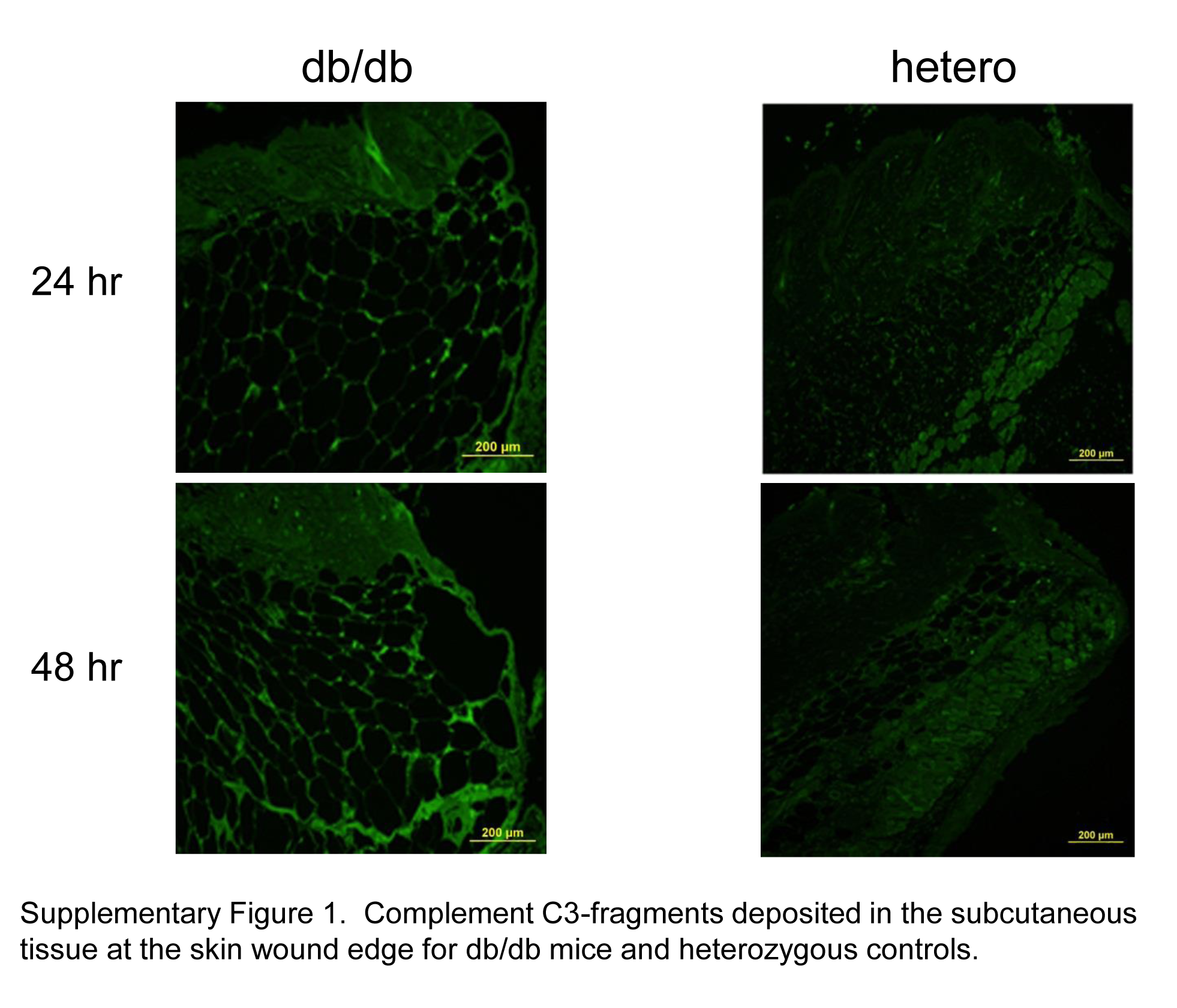

Supplement: S1 Fig — Representative micrographs showing complement C3-fragments deposited in the subcutaneous tissue at the skin wound edge for db/db mice and heterozygous controls. (TIF) [file pone.0170500.s001.tif]

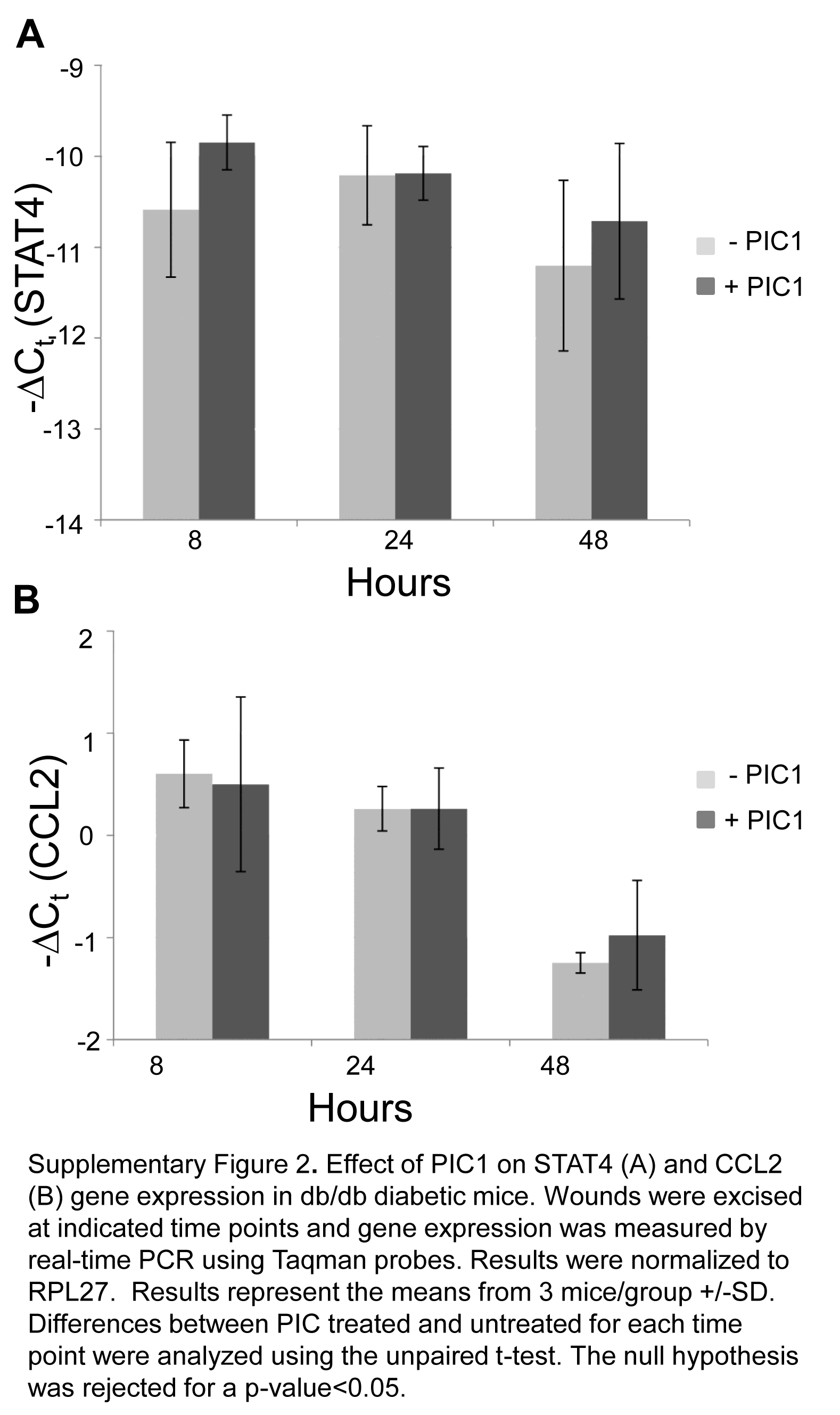

Supplement: S2 Fig — Wounds were excised at indicated time points and gene expression was measured by real-time PCR using Taqman probes. Results represent the average from 3 mice/group ±SEM. Differences between PIC1 treated and untreated wounds for each time point were analyzed using the unpaired t-test. The null hypothesis was rejected for a p-value <0.05. (TIF) [file pone.0170500.s002.tif]
